# Maneuvering on non-Newtonian fluidic terrain: a survey of animal and bio-inspired robot locomotion techniques on soft yielding grounds

**DOI:** 10.3389/frobt.2023.1113881

**Published:** 2023-06-06

**Authors:** Simon Godon, Maarja Kruusmaa, Asko Ristolainen

**Affiliations:** Centre for Biorobotics, Department of Computer Systems, Institute of Information Technologies, Tallinn University of Technology, Tallinn, Estonia

**Keywords:** multiphase environment, soft grounds, yielding grounds, animals, robots, bio-inspiration, locomotion, non-Newtonian fluid

## Abstract

Frictionally yielding media are a particular type of non-Newtonian fluids that significantly deform under stress and do not recover their original shape. For example, mud, snow, soil, leaf litters, or sand are such substrates because they flow when stress is applied but do not bounce back when released. Some robots have been designed to move on those substrates. However, compared to moving on solid ground, significantly fewer prototypes have been developed and only a few prototypes have been demonstrated outside of the research laboratory. This paper surveys the existing biology and robotics literature to analyze principles of physics facilitating motion on yielding substrates. We categorize animal and robot locomotion based on the mechanical principles and then further on the nature of the contact: discrete contact, continuous contact above the material, or through the medium. Then, we extract different hardware solutions and motion strategies enabling different robots and animals to progress. The result reveals which design principles are more widely used and which may represent research gaps for robotics. We also discuss that higher level of abstraction helps transferring the solutions to the robotics domain also when the robot is not explicitly meant to be bio-inspired. The contribution of this paper is a review of the biology and robotics literature for identifying locomotion principles that can be applied for future robot design in yielding environments, as well as a catalog of existing solutions either in nature or man-made, to enable locomotion on yielding grounds.

## 1 Introduction

The last decades have witnessed a rapid advancement of robotics applications from well-structured and defined industrial environments into an unstructured and dynamic external environment (Bruzzone and Quaglia, 2012; Rubio et al., 2019). Robots in the real world can move on solid ground as well as in the air and water, and consequently, researchers and engineers have developed terrestrial, aerial, and underwater robots (Aguilar et al., 2016). However, robots still cannot access all types of natural environments (Bruzzone and Quaglia, 2012; Aguilar et al., 2016; Rubio et al., 2019).

Soft yielding substrates are materials that significantly deform under the application of stress and do not recover their original shape when stress is released. These materials can present a yield stress under which they do not undergo plastic deformation, but once pressure exceeds the yield stress, the material flows and undergoes irrecoverable, plastic deformation (Balmforth et al., 2014; Coussot, 2014). Such materials are common in nature: soils are a mixture of different particles such as gravels, sands, silts, and clays (depending on the particle size) mixed with air, water, and organic matter (Barnes, 2016). Mud is such a medium with high water content. Sand, snow, and leaf litter are other examples of such materials with varying properties (Wong, 2009; Balmforth et al., 2014; Barnes, 2016).

The problem of locomotion in deformable grounds is important to solve because it would facilitate new robotic applications such as search and rescue in wet forests, muddy fields, avalanches, and mudslides (Schneider and Wildermuth, 2016); for the agricultural vehicles operating on wet soils (e.g., rice fields) (Duckett et al., 2018; Oliveira et al., 2021); for exploration or excavation of materials (e.g., wood and ore) with a minimal environmental impact (Billingsley et al., 2008; Lopes et al., 2020); for environmental monitoring in high-biodiversity areas (e.g., river estuaries, bogs, and shores) (Dunbabin and Marques, 2012); or for extra-terrestrial exploration (Li and Lewis, 2022).

The field of terramechanics (Bekker, 1960; Wong, 2009) covers interactions between vehicles and natural grounds from a traction perspective. It covers the theory of vehicles moving with wheels or tracks on mud, sand, or soil. However, as we demonstrate in this article, there are very different ways of moving on soft deformable grounds, and terramechanics covers only one of these, which is the method used by all wheeled and tracked vehicles.

Well-known manned vehicles and some robots use wheels and tracks to move in these environments up to a limit (Bruzzone and Quaglia, 2012). Usually, they are large and heavy enough to deform the medium and gain traction from the solid bottom under the loose substrate if the medium is non-homogeneous and from the unyielded buried substrate, otherwise. Yet, this can fail if the medium is too deep and weak because actuators cannot generate sufficient tangential forces to move a potentially buried body. Some robot prototypes are addressing the challenge of traversing yielding terrains. Some quadruped (Raibert et al., 2008; Bagheri et al., 2017) or hexapod robots with rigid (Li et al. (2013); Li et al. (2009)) or adaptable legs (Liang et al., 2012) have been shown to be capable of negotiating those terrains. The SeaDog robot uses spoke wheels, which combine some advantages of wheels and legs (Klein et al., 2012), and the ePaddle robot uses wheels with expandable paddles (Shen et al., 2018). Undulatory robots mimicking worms (Sfakiotakis et al., 2016), snakes (Marvi et al., 2014), or lizards (Maladen R. D. et al., 2011) are other examples. Unstructured environments have also been negotiated by crawling robots such as a sea-turtle robot (Mazouchova et al., 2013) and a mudskipper robot (McInroe et al., 2016). A razor clam robot was designed, capable of digging through mud (Winter et al. (2014)). Some researchers designed screw-based robots, having either two screws (Nagaoka et al., 2010b) or four (Lugo et al., 2017). Recently, some robots were designed to challenge the granular lunar terrain (Shrivastava et al., 2020) by combining wheels and walking gaits. On a different scale, a sperm-inspired robot has been built, moving at low Reynolds numbers (Khalil et al., 2016). Aguilar et al. (2016) introduced the field of robophysics which consists of studying the motion of moving systems by complementing the study of complex robots with simplified robotics experiments and simple theoretical models. It provides a review of research that has already helped with the understanding of unstructured environments.

Bioinspiration and biomimetics seems to be the most commonly used paradigm for developing robots for yielding environments: the majority of the aforelisted examples explicitly claim to be inspired by animal locomotion. Typically, they mimic a specific animal or an aspect of the locomotion of a specific animal. Bio-inspired robotics can be used in two ways: one can take inspiration from a known working solution taken from nature to make a robot particularly well-suited for an environment, thereby benefiting robotics. It can also be used the other way around: one can make a robot that mimics a living being to understand some of its working principles, thereby benefiting biology (Gravish and Lauder, 2018). The concept of biomimetics and bioinspiration is a specific case of a problem-solving technique called design-by-analogy (Verhaegen et al., 2011) and often has its focus on biomechanics (Goel et al., 2017). The analogy between biology and engineering can be derived at different levels, but the decisive phase is the mapping from the problem domain to the solution domain using the most appropriate level of abstraction (Vincent et al., 2006; Verhaegen et al., 2011). It is critical to find an appropriate abstraction level for the task when using biomimetics (Nagel et al., 2010), and a higher level of abstraction allows for the creation of links between different principles that are independent of the form or behavior of biological entities (Mak and Shu, 2004). When abstract principles have been derived, they can be transposed to technology and the abstraction level can be lowered to reach technological solutions for which direct mimicry of biology–technology may not be feasible (Baumeister et al., 2013; Fayemi et al., 2017).

A majority of the robotics literature we analyzed chose to mimic the anatomy of an animal, its locomotion, or a feature of its body or behavior. Through mathematical modeling, some researchers have begun to unify these principles of locomotion in dry granular media. For example, Astley et al. (2020) reviewed the principles and mathematical modeling of limbless locomotion in dry sand, Zhang and Goldman (2014) demonstrated the applicability of resistive force theory (RFT) in dry granular media, and Hosoi and Goldman (2015) proposed a mathematical modeling technique for digging and burrowing in granular media and described four regimes based on the size of the animal and the inertial number. Other works also address different media. For example, Dorgan (2015) established the mechanisms of subterranean locomotion in dry and cohesive media, and Aguilar et al. (2016) proposed using mathematical modeling, simulations, and experimental validation as a systematic approach to locomotion in a variety of environments such as air, water, hard ground, or cluttered environments and goes beyond the field of yielding media. There, locomotion in yielding materials is treated from an anatomical point of view and describes the modes of locomotion as a legged, flipper-based, sand-swimming, or two-anchor mechanism.

The very active work on modeling presented previously is a necessary effort to understand the mechanisms of locomotion in yielding media. However, while reviewing the research work on biology, we found that, in the vast majority of cases, mathematical modeling of the animals’ locomotion was not available. To enable their study, we propose turning to a higher abstraction level where locomotion can be studied from a qualitative point of view, such as the higher level prescribed by the biomimetics methodology.

In this paper, we review biology literature addressing locomotion in soft, deformable environments and propose some general principles for designing robots for those terrains. We use abstraction at the level of general mechanical principles of the deformable medium to derive engineering goals. We also categorize the existing robotics literature and demonstrate that some of those principles have been explicitly or implicitly already used in robots or other vehicles, and since they are sufficiently general, the resultant design does not necessarily need to be explicitly bio-inspired. This overview aims to offer a systematic approach, general guidelines, and design targets for developing better vehicles for soft yielding environments. The higher abstraction level used in this overview enables drawing parallels between locomotion strategies that may, at first, seem distant from each other. As discussed previously, the use of a high abstraction level such as the one we chose is advocated by the biomimetics methodology. With this paper, we aim at providing future researchers in this field with a tool to pass from the problem domain to the solution domain and find an adapted solution for their application related to locomotion in yielding environments. In addition to this engineering tool, we believe this classification can aid in the discovery of links between locomotion strategies and provide hints for future research into the unification of locomotion theories in soft yielding materials. Last, the present paper will be a useful catalog of possible sources of inspiration for locomotion in soft media. The principles and strategies of locomotion presented in this paper could be further modeled and experimented with using and extending the theories presented previously, for example, through the robophysics framework.

## 2 Background on non-Newtonian, yielding materials

Yielding materials are a subset of non-Newtonian fluids, which are fluids that exhibit shear stress that is not proportional to the shear rate. Over the last century (Alderman (1997); Denn (2004) for overviews), researchers have identified a wide range of complex behaviors exhibited by non-Newtonian fluids, including shear-thinning, shear-thickening, Bingham plastics, and viscoplastic fluids (Bingham (1917; 1922); Herschel and Bulkley (1926); Oldroyd (1947); Metzner and Reed (1955); Metzner (1956); Schowalter (1960)). Rheology, which is the study of the flow and deformation of matter, is a fundamental discipline that bridges the study of non-Newtonian fluid mechanics and the theory of plasticity (Barnes et al., 1989; Larson, 1999). It provides a framework for understanding and quantifying the complex behaviors of non-Newtonian fluids, as well as other complex materials beyond fluids, such as polymers, gels, pastes, and muds. This understanding of complex materials is essential to comprehend the behavior of soil, which is a complex substrate made up of four components: minerals, air, water, and organic matter. These components can be present in varying proportions depending on precipitation, proximity to water bodies, or compression of the material. Depending on the particle size of minerals, the soil particles can be classified as clay (smallest), silt, sand, or gravel (largest). When the water content is very high, the soil behaves as a liquid. When the water content decreases, the soil behaves as a plastic or viscoplastic material and is solid when the water content is low (Barnes, 2016). The plastic behavior means that above a certain stress, called yield stress, the material deforms and does not return to its original shape. The smaller the particles, the lower the permeability to water, and the more prominent the plastic behavior is.

The stress applied on the material can be computed with the von Mises criterion (1):

$$\sigma =\sqrt{\frac{{\left(\sigma_{1}-\sigma_{2}\right)}^{2}+{\left(\sigma_{2}-\sigma_{3}\right)}^{2}+{\left(\sigma_{3}-\sigma_{1}\right)}^{2}}{2}},$$ (1)

where *σ*_*i*_ denotes the stresses in each principal direction and is calculated as in Eq. 2, and *σ* denotes the von Mises stress. Above a certain value called “yield stress,” the material will undergo plastic deformation and flow. For non-cohesive materials like sand or dust, the yield stress is negligible, and the material starts to flow as soon as it is pressed upon.

$$\sigma_{i}=\frac{F_{i}}{S_{i}}.$$ (2)

In Eq. 2, *F*_*i*_ is the force in direction *i*, and *S*_*i*_ is the contact area in the same direction. Forces can be either due to the weight of the body or due to acceleration.

Viscoplastic behavior exhibits both solid and fluid properties, and material deformation is also affected by the rate of stress (Kutter and Sathialingam, 1992; Balmforth et al., 2014; Coussot, 2014). A large selection of models exists for muddy/clayey/sandy soils, but a unifying model is still to be found (Liingaard et al., 2004; Karim and Gnanendran, 2014). Soft yielding grounds are manifold and can present different properties, depending on whether they are cohesive, such as mud and wet sand, or not cohesive, such as dust or dry sand. More specifically, contrary to non-cohesive yielding grounds, cohesive grounds have a yield stress and are viscous (Augustesen et al., 2004; Omidvar et al., 2012; Mishrai et al., 2016; Yang et al., 2017).

To move, a body must exert forces on the environment. The environment, in turn, generates a counter-reaction force that propels the body. On a solid flat ground, if the body stands still, the force on the ground, called the ground reaction force, has only a normal component to compensate for the weight of the body. When the body moves, it must exert a force parallel to the ground, which is the horizontal component of the ground reaction force and is limited by the friction coefficient of the ground/body couple. When the ground is soft, it can deform under the weight of the body, and the maximum force is also limited by its shear resistance. The deformation of the material causes energy losses that make locomotion more difficult as more work is required (Lejeune et al., 1998). The work W exerted on a deforming medium is defined as in Eq. 3, where z is the depth of intrusion and F the force as a function of z.

W=∫Fdz. (3)

The body will move forward when it generates sufficient forces *F* in the direction of movement over some time *t* (mechanical impulse *I*) to move its mass *m* at a certain speed *v* (to gain momentum):

I=∫Fdt=mΔv. (4)

When the soil is yielding, the applied force is limited by the yield force in the direction of movement, reducing the impulse and slowing the body down. To reduce sinkage into the media, robots or animals have to apply reduced pressure. When sinkage already occurred, the animals or robots have to deal with moving in a deformable material, where, depending on the material, stress, rate of stress, and depth of insertion can all play a role (Terzaghi et al., 1996).

**FIGURE 1:**
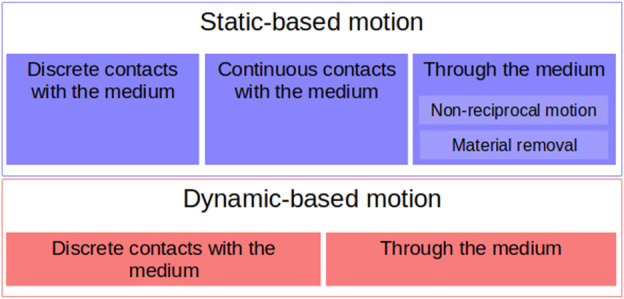
Classification of the principles and strategies of locomotion in yielding grounds.

Given that soft yielding materials exhibit different behaviors depending on how one interacts with them, there are two different ways of moving in/on them. When moving on a yielding substrate, frictional hydrostatic forces dominate at low speeds, while the inertial hydrodynamic response of the material dominates at high speeds (Qian et al., 2012). There are, thus, two ways to move on or through a yielding material: using the material’s static properties or using its dynamics properties (Figure 1).

There are primarily two ways to move using the static properties of the medium, as shown in the top part in Figure 1. The first way is to not exceed the yield stress of cohesive materials or deform them plastically so that the resulting surface can be used as a static support against which to generate thrust. When we do not consider dynamics, the vertical equilibrium is maintained between hydrostatic-like pressure on the immersed volume of the body and weight. We refer to hydrostatic-like pressure as a pressure that increases proportionally with depth (Aguilar and Goldman, 2016). It has been shown that different flowable materials, when stepped upon, generate a reaction force that is almost proportional to the depth of intrusion (Sharpe et al., 2013; Godon et al., 2022; Ma et al., 2022). In the horizontal direction, thrust can be generated by relying on the cohesion and adhesion of the medium particles with the body. Additionally, if plastic deformation is carried out, the body can push horizontally against the unveiled surface of the material.

In the second case, the moving body uses the dynamic fluid properties of the medium (see the bottom part of Figure 1). To move dynamically on/in a fluid, a body has to transfer momentum to the fluid so that the fluid reacts by providing thrust, as per the principle of the conservation of momentum. There are three main momentum transfer mechanisms in fluids: drag (pressure and friction drags), lift, and acceleration reaction forces. However, in friction-dominated media, lift cannot be used for propulsion (Webb, 1988), and pressure drag is another manifestation of inertial forces (Vogel, 2020). This leaves two mechanisms for movement through a frictional fluid. The first, friction drag, can be used in static locomotion and is also a necessary component of pressure-drag-induced inertial forces. The second, acceleration-related force, takes advantage of propelling fluid particles in the opposite direction of the desired thrust. In nature, this is usually performed in the fluid by oscillations or undulations of the body and appendages in fish, ducks, or seals (Sfakiotakis et al., 1999; Vogel, 2020), but can also be performed at the surface by slapping the fluid. In this rapid motion regime, the forces exerted by the fluid depend quadratically on velocity. We refer to this as the hydrodynamic-like principle (Aguilar and Goldman, 2016) because of its similarities to the hydrodynamic principles of propulsion exerted in fluids (Webb, 1988; Sfakiotakis et al., 1999; Vogel, 2020).

As opposed to moving on a deformable soft media, dynamic motion through such medium is hindered by high friction drag and form drag. One solution to reduce drag is to use fluidization. Fluidization is the process by which material particles are given a sufficient velocity so that the granular material (cohesive or not) behaves as a fluid. This can be performed by injecting a fluid through the materials, which will generate a form drag on the particles and reduce the interparticle stress. Fluidization can also be enhanced by vibrations in a granular material (Zik et al., 1992; Xu and Zhu, 2006). Fluidization is mostly used to move through the substrate to facilitate progression and is hence typically observed in animals digging or burrowing in granular media (Hosoi and Goldman, 2015).

Thus, locomotion on non-Newtonian yielding ground is different from that on solid ground or in water. To move on/through such materials, animals have developed a wide range of locomotion strategies. These strategies are explored and categorized in this paper, and existing robots are categorized accordingly.

## 3 Methods

This paper aims at answering the following questions:

- How to classify the modes and mechanisms of locomotion in natural, yielding environments?

- What physical principles are present in nature for locomotion in yielding environments?

- Do those modes and mechanisms share any common physical principles that are abstract enough to be applied to robots including non-bio-inspired robots?

To answer these questions, we conducted a review of biology research, focusing at first on animal locomotion in soft yielding environments. We identified relevant research papers and classified them based on material behavior and locomotion mechanisms. We have also mapped existing robots into the presented principles, demonstrating that successfully developed solutions are designed implicitly or explicitly against those principles and suggesting that defining those principles as design targets will help develop better vehicles faster and easier.

Our integrative literature review on the biology of locomotion in deformable environments used established methodologies outlined in Torraco (2005); Snyder (2019). We utilized relevant keywords associated with the themes of locomotion, animals, and soft environments (Table 1) to search various online databases such as Google Scholar, IEEExplore, ACM Digital Library, Science Direct, Web of Science, Wiley Online Library, Scopus, CiteSeerX, SpringerLink, PNAS, and PLOS One. Our search terms involved combining at least one word from the first column with at least one word from the second column in Table 1 to create different keyword combinations (e.g., “animal locomotion in multiphase environments,” “walking fish on mud,” and “legged locomotion on low resistance ground”). Additional papers were identified from the references in the documents. To ensure inclusiveness, all papers mentioning any of the selected keywords were selected at first. Then, analysis of the main topic of the documents led to keeping only those addressing locomotion from a mechanical perspective. For instance, documents discussing the evolution of the genome, neural control, muscle control, fish swimming near the surface, or an evolutionary analysis of anatomy were excluded. The documents were then divided into categories based on the main topic, and some categories were disregarded as being out of scope for this literature review (e.g., addressing the effect of viscosity on swimming or the burrowing patterns of crustaceans). The most representative documents of each category constitute the corpus of this article and were organized based on the mechanical principles and locomotion strategies employed by the animals they describe. Papers relating to robotic analogs were included at a later stage.

**TABLE 1:** Keywords used for the literature search.

About the animal/action	About the environment
Amphibious, animal, benthic, boring, burrowing, crawling, fish, fossorial, legged, locomotion, motion, semi-terrestrial, walking	Clay, flowable, ground, intertidal, low resistance, mangrove, mud, multiphase, sand, slurry, substrate, soft, unstructured, viscoplastic, weak ground, wet granular media, yielding, yield stress

We, then, analyzed the locomotion used by each species and robot and abstracted it to understand how and where the forces are generated. We discovered that even when forces were generated using different body parts, motions, speeds, and material properties, they could be classified into two categories based on whether they used the static or dynamic properties of the yielding media. Then for each mechanical principle, the interaction type could be generated using different strategies, which mainly differed depending on the nature of the contact between the body and the medium. Furthermore, under this second level, we discuss the animals’ specific means of locomotion, describing which body parts are used, how they are used, and the special features facilitating the means of locomotion.

In the next sections, the locomotion strategies classified according to the mechanical principle will be more closely described, along with a description of the specific animal locomotion patterns and references to the existing robotic analogs.

In the following sections, the animals’ and robots’ means of locomotion on soft yielding grounds will be categorized into the two following interaction types: those using the static properties of the material, mainly using friction-based hydrostatic pressure, and those using the dynamic properties, mainly inertia.

## 4 Statics-based movements

Statics-based movements use the hydrostatic-like pressure in the medium to counter gravity, friction forces, and material cohesion to generate forward impulse. We observed that the highest level of distinction that could be made was based on the spatial distribution of the contact between the body and the media. Three different strategies were observed:

• Discrete contacts with the medium
• Continuous contact at the surface of the medium
• Immersion through the medium

The two first strategies imply compensating the body’s vertical and forward momentum on the ground by achieving static equilibrium between the deformable material and the body. There are two possibilities: the stresses exerted by the body on the medium are less than or equal to the medium’s yield stress. In most cases, unless the animal is very light-weighted, or the ground is almost solid, the material first yields, and then solidification occurs when pressure drops below the yield stress: at first contact, only a minor portion of the body, for example, a foot, touches the ground, and the pressure is very high. The surface yields, the foot sinks into the soil until a sufficient surface area touches the substrate to distribute the efforts, and the relative pressure is reduced to the yield stress. Resistive force increases linearly with depth: the deeper the appendage sinks into the substrate, the higher the pressure, and the more the substrate resists intrusion. Only when solidification has occurred, the animal can use the substrate as a static support. Yet, at this stage, the material has deformed, required work, and made locomotion harder. There are two different possibilities to reduce sinkage: reduce the pressure exerted on each weight-bearing body part and reduce acceleration-related forces.

Animals use different strategies to achieve such results: using a large number of legs, using large feet surface areas, lying on a large portion of the trunk, using the tail to increase the contact area, or reducing forces by moving slower. In the third strategy, through the medium, the body is inside the substrate, and hydrostatic-like pressure does not need to compensate for the weight of the body outside the material. There, the challenge is rather to generate forces using non-reciprocal movements because the friction-dominated environment dissipates energy, and therefore inertia (Purcell, 1977; Vogel, 2020).

### 4.1 Animal locomotion

#### 4.1.1 Discrete contacts with the medium

Ungulates (large mammals with hooves) step on yielding substrates with hooves. The relatively small surface area of the hoof is initially insufficient for compensating their weight without yielding the substrate. As a result, the yield stress of the material is exceeded and the leg starts to sink; the foot of the animal sinks into mud until it encounters something harder or until the hydrostatic-like pressure compensates for the weight. One interesting feature observed in ungulates is the passive enlarging of their feet when stepping on yielding material. Not only do the different toes move apart from each other, but also in some cases, digits are located higher at the back of the foot. The rear digits also passively extend outward when the leg sinks into the yielding substrate and passively retract when the leg is removed from the substrate. Two studies were found in the literature about locomotion of cows in slurry or sand (Phillips and Morris, 2000; Telezhenko and Bergsten, 2005). When in a not too deep layer of slurry, cows reduce their stride frequency to compensate for the difficulty of penetrating and retracting the leg through the slurry. Simultaneously, they increase their stride length because the risk of slipping is reduced when the animal has a strong foothold in the slurry (Phillips and Morris, 2000). The same increase in stride length was observed when walking in sand compared to hard ground (Telezhenko and Bergsten, 2005). The foothold providing additional traction has been experimentally studied on a test bed in granular materials (Yeomans et al., 2013).

**FIGURE 2:**
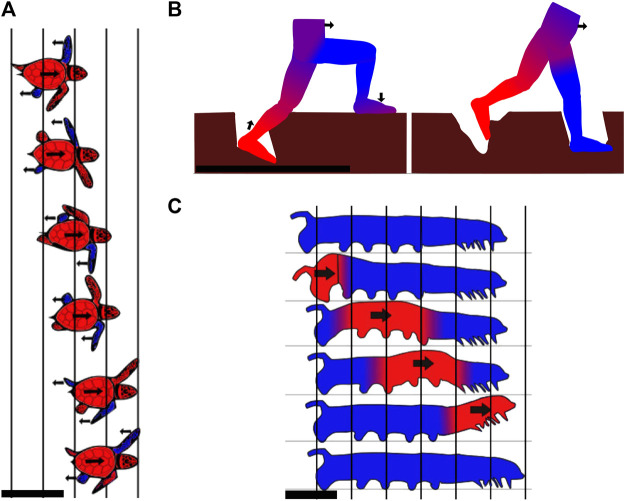
Example gaits of animals using static-based locomotion with discrete contacts. **(A)** Stepping pattern of a quickly moving hatchling turtle using only its four appendages. The blue parts are the flippers that are supporting against the ground, while the red parts are those moving forward. Two flippers are pushing backward/downward against the sand at the same time, while the other two are in the recovery motion and brought forward in the air. Top view, scale bar is 5 cm. Drawn according to Lutz and Musick (1996). **(B)** Illustration of a person walking through mud. One leg sinks and deforms mud until the hydrostatic pressure compensates for the human’s weight. When the leg is bearing the entire weight, the other leg is retracted and placed further. The mud undergoes plastic deformation and does not fully recover its original shape. The blue parts are flippers supporting against the ground, while the red parts are those moving forward. Side view, scale bar is 1 m. **(C)** Movement cycle of the caterpillar. The moving parts are highlighted in red and the static parts in blue. Progression of the body happens outside of the medium while the feet make discrete contacts with the ground to generate the reaction forces. Side view, scale bar is 1 cm.

Similarly, it was found that in the case of hermit crabs, bigger individuals ran faster on beach sand by increasing stride length, but not frequency (Herreid and Full, 1986). Other research showed that basilisk lizards also increase their speed on sand by solely increasing their stride length (Bagheri et al., 2017). Salamanders use undulations of their body to take larger steps (Edwards, 1989) and walk on mud using a gait (Aydin et al., 2017) which keeps three legs on the ground to reduce the pressure on the substrate. Tiger salamanders additionally increase the surface area of their feet contacting the ground when the medium is compliant, but it is not clear whether this is a passive process due to surface softness or an active mechanism (Vega and Ashley-Ross, 2020). The same phenomenon was observed in hatchling turtles using the alternating gait in which diagonally opposed flippers push on the media. During the stroke, the plastron is lifted off the ground to reduce drag, and the flippers are oriented perpendicularly to the propulsive force to decrease soil deformation. An illustration of the hatchling turtles using the alternating gait is shown in Figure 2A. The Uma lizard has fringed toes that may facilitate locomotion on sand. These fringes increase the surface area, which, in turn, increases frictional forces and decreases pressure (Carothers, 1986). However, a more recent study disagrees and reveals that these fringes have an advantage in burrowing (Zheng et al., 2020). Generally, it has been shown that lizards living in sandy areas have longer feet relative to their size (Kohlsdorf et al., 2001). Additionally, lizards with larger feet could passively reduce their penetration ratio and maintain better performance on flowable grounds. For many lizard species running on sand, it was demonstrated that the acceleration was lower on sand than on less compliant surfaces (Vanhooydonck et al., 2015). The same observation has been made on humans. It can be explained in part by the mechanical work lost when deforming the sand, and in part by decreased muscle and tendon efficiency in positions reached by the feet (Lejeune et al., 1998). Moreover, similar to what was observed for cows, lizards, and crabs, humans increase their stride length on very compliant surfaces (McMahon and Greene, 1979). Figure 2B depicts a human stepping on yielding mud.

Other large animals, such as elephants or camels, also increase the surface area of their feet when stepping. Fat pads in camels exhibit viscoelastic behavior and expand more as more pressure is exerted on them. This pressure-dependent expansion enables pressure on the ground to be independent of velocity. It also acts as a dampener, reducing the loading rate and peak force. Additionally, fat pads reduce localized pressure build-up, which enables traversing rocky terrains without damaging the foot (Clemente et al., 2020). Similarly, elephants can also walk on the waterside or desert sand thanks to their fat pads (Weissengruber et al., 2006), which have viscoelastic properties and enable them to absorb shocks and adapt to the ground (Panagiotopoulou et al., 2012).

Many light-weighted legged animals are also described walking on deformable grounds. For these animals, their weight is low enough or their feet surface area is sufficient to reduce the stress exerted on the ground, hence reducing deformations. For example, arthropods are lightweight and have six to hundreds of legs. They are often found in areas where the ground is soft, for examples, crabs on the seaside, scorpions in the sand, and centipedes in forest soils (Foxon, 1936; Trueman, 1983; Faulkes and Paul, 1997; Herreid, 2012; Kuroda et al., 2014). Caterpillars (Trimmer et al., 2006) and inchworms (Plaut, 2015) are also very light-weighted animals that use several pairs of paws moving in an undulating pattern. Even though such larvae’s natural habitat is not directly mud or sand, they can often be found moving with ease on such substrates. An illustration of the caterpillar gait can be found in Figure 2C

One common feature we can notice in all these animals is the presence of mechanisms to reduce foot pressure: large paws, spreading/retracting digits (anisotropy), long fingers or a large number of legs, soft feet absorbing shocks, and spreading weight or gait lowering pressure on feet. It is worth noting that many animals we find in the desert or along the watersides have webbed feet or long fingers, increasing the surface area: Gila monster, shovel-snouted lizard, web-footed Namib dune gecko, ducks, beavers, and otters. For animals walking in such media, keeping a relatively low speed was observed to be a consistent behavior. This could be to reduce frictional losses as well as acceleration-related forces that could further yield the substrate and plunge appendages deeper into it (Li et al., 2012).

#### 4.1.2 Continuous contacts at the surface of the medium

The strategy presented in this section consists of keeping the main body constantly in contact with the ground. Crawling animals use a large part of their body to lie down on the ground and use appendages or undulations of the body to generate forces parallel to the ground.

**FIGURE 3:**
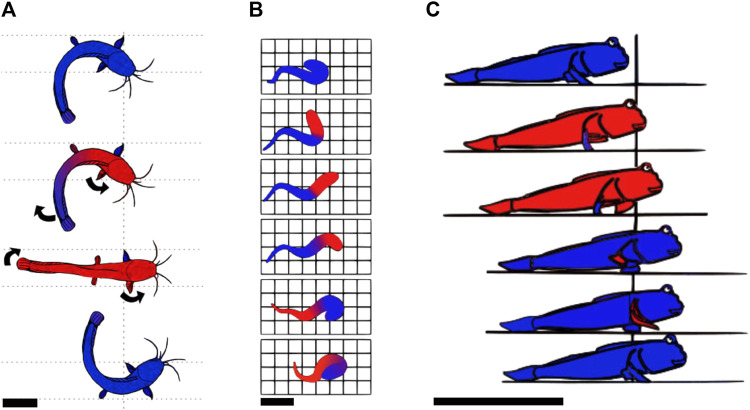
Example gaits of animals using a static-based locomotion with continuous contact. **(A)** Locomotion of the *Clarias* on land according to Johnels (1957). The red parts are those progressing, while the blue parts are those pushing against the ground or are static. The *Clarias* anchors one pectoral fin in the ground and uses its tail to pivot its body around the anchored fin. It then anchors the second pectoral fin and repeats the cycle on the other side. Top view, scale bar is 10 cm. **(B)** Representation of the locomotion pattern of the lungfish on land. The red parts represent those that are progressing, while the blue ones represent those that are pushing/pulling on the soil. The lungfish propels its head by pushing on its tail and anchors its head in the ground. Then, it brings the tail closer to the head, using the head as an anchor and starts a new cycle. Top view, scale bar is 10 cm. Drawn according to Horner and Jayne (2014). **(C)** Locomotion pattern of the mudskipper using the alternating tripod system. The red parts are moving forward while the blue ones are not. The mudskipper uses its pectoral fins to generate lateral forces on the ground and rests on the pelvic fins during the recovery motion of the pectoral fins. Throughout the entire locomotion cycle, the tail is trailing on the ground. Side view, scale bar is 10 cm. Drawn based on HARRIS (1960).

Crawling is a mode of locomotion often used by animals living in water and occasionally venturing onto the shore. This suggests that this mode of locomotion is unlikely to be optimized for land locomotion, but rather a way for bodies adapted to aquatic environments to move on land. A large number of these animals are fish. Fish move in water using axial undulations. Some evolved limbs and invaded land over time (Amaral and Schneider, 2018), resulting in locomotion strategies that combine body undulations and limbs. For example, *Polypterus senegalus* is a fish which uses its fins along with longitudinal rotations of its body to generate motion, while the body is resting on the ground (Standen et al., 2016). Climbing perches, lungfishes, and *Clarias* all use a similar crawling-based locomotion on land: they all lay their entire body on the ground, distributing their weight over a large surface area, and then anchor a part of their body by plastically deforming the ground. Then, they apply lateral force to this anchor to propel themselves. The climbing perch uses this principle by planting the detachable sub-operculum into the ground to use it as a pivoting point for propelling its body forward (Davenport and Matin, 1990). Using the same strategy, the *Clarias* plant their fin (Johnels, 1957) and the lungfish plant their crane (Horner and Jayne, 2014). Figure 3A presents *Clarias* moving on land using this strategy, and Figure 3B presents the crane anchoring of lungfish. In all those cases, the plastic properties are used to plant the anchor into the mud, and the frictional properties are used when pulling. This way of combining appendages and body undulations is described as axial-appendages; one of the three modes of locomotion of fish on land (Pace and Gibb, 2014), the other two being undulations or appendages only. These locomotion patterns are probably the most primitive ways of moving on soft media as they have been adopted by the first fishes invading land (Amaral and Schneider, 2018).

When crawling, animals such as the mudskipper (HARRIS, 1960; Van Dijk, 1960; Pace and Gibb, 2014) or hatchling turtles during their symmetric gait (crawling simultaneously with both front flippers) (Mazouchova, 2012) propel themselves by pushing backward/downward with their flippers, while leaving a large part of their body dragging on the ground. In the propulsion phase of the mudskipper, the fins are placed flat on the surface of the ground to obtain the maximum traction possible and take advantage of friction to exert lateral forces. During the hatchling turtle’s propulsion phase, sand is solidified by positioning the flipper normal to the direction of efforts, increasing the effective surface area, and then used to propel the body. While the limbs are in a swing phase (moving without touching the ground to prepare for another stroke), the animal lies on a larger portion of the body, which enables the material to not significantly deform. The larger portions of the body used are the pectoral and caudal fins for the mudskipper and the carapace for the turtle. The crawling locomotion pattern of the mudskipper is shown in Figure 3C.The mudskipper has been observed to use its tail more and more as the steepness of the incline increases (McInroe et al., 2016). This mechanism also prevents back slippage.

The remaining crawling animals found in the literature move on soft ground by undulating their bodies. Gastropods move on such substrates while not yielding them (Trueman, 1983). Their light weight, together with the large surface area of their foot, enables very little pressure to be exerted on the substrate. The body of gastropods continuously stays in contact with the ground, and peristaltic-like undulations of the foot generate propulsion forces. Snakes use different locomotion patterns, all consisting of undulations of different sorts. Their locomotion patterns consist of keeping a large part or all of the body on the ground, and then they slide the body by pushing on natural obstacles or lumps of sand that they formed themselves during sidewinding locomotion by deforming and solidifying the sand surface (Wake, 2001). In the more challenging case when no obstacles are available to push against and the substrate is too hard to be deformed, scale anisotropy and weight distribution help snakes to move on a substrate (Hu et al., 2009); this is used in concertina and rectilinear locomotion patterns (Jayne, 1986; Wake, 2001).

Leeches and worms on mud use peristaltic undulations, where the parts in contact with the ground are large enough so that friction resists backward movement while the rest of the body is moving forward (Dorgan, 2010; Kristan, 2019). The polychaete *Nereis virens* also uses body undulations from the back to the front in combination with a rowing pattern of the legs (La Spina et al., 2007).

Seals are the only example found in the literature of a large animal moving on such substrates (sand and gravel) using undulations (O’gorman 1963). The seal’s entire body is in contact with the ground and undulates in the sagittal plane at slower speed and in the frontal plane at greater speeds. Similar to all animals in this section, keeping a large portion of the body always on the ground reduces the seal’s sinkage.

#### 4.1.3 Through the medium

In this third static-based strategy, animals move through the media. There are two ways to do this: either by deforming the body and using non-reciprocal motion or by removing the material, thereby removing the frontal motion resistance.

##### 4.1.3.1 Non-reciprocal motion

**FIGURE 4:**
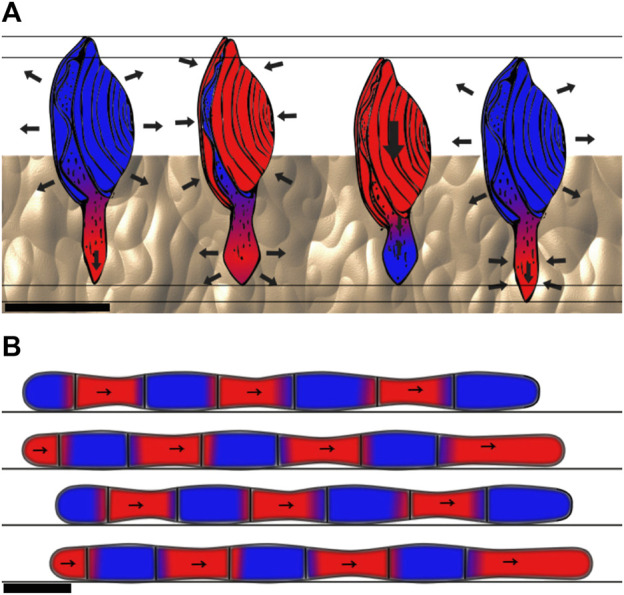
Example movements of animals using non-reciprocal static-based locomotion. **(A)** Locomotion cycle of a clam. The moving parts are specified with red color and the static ones with blue. The digging pattern of the clam is based on a dual-anchor: the shell is expanded and applies forces on the surrounding medium, which anchors the shell in place. Then, the foot, shaped in a thin, elongated form, penetrates the substrate further. The foot is then expanded and the shell closes so the foot becomes the anchor, and the foot pulls the shell. Then, the shell is expanded again for a new cycle. Side view, scale bar is 1 cm. Schematics is based on Dorgan (2015); Trueman (1983). **(B)** Worm using peristaltic motion. The red parts are those progressing, while the blue ones are anchored to the soil. The peristaltic motion consists of waves of contraction and release of circular and longitudinal muscles. Parts of the body are expanding and generating forces against the ground, while the body parts that do not touch the substrate are progressing. Side view, scale bar is 1 cm.

In a low Reynolds number regime, the viscous forces dominate the inertial forces. In this case, locomotion cannot rely on the principle of giving momentum to a fluid because viscous forces dampen any inertia, and animals have to use locomotion strategies based on non-reciprocal motion (Purcell, 1977; Pak et al., 2015). Sperms use flagella while moving in low Reynolds number environments. To break the symmetry and enable propulsion at low Reynolds numbers, the flagella oscillates from side to side, bending in a chiral shape (Friedrich et al., 2010). This strategy is also typically used by clams, which move through the medium using a dual-anchor mechanism in which a part, either the shell or the foot, is shrunk and pushed through the medium, while the other is expanded and *vice versa*. This strategy of changing the shape of different body parts during the motion or the anchoring phase creates a non-reciprocal motion that enables one to move without making use of the medium’s inertia. This behavior can be observed in Figure 4A.

Burrowing eels have been observed to burrow into the bottoms of water bodies using a high slip factor (ratio of undulation wave speed to locomotion speed). This means that the undulating wave of the body travels at almost the same speed as the body and that the substrate behaves as a solid. Consequently, little energy is lost in substrate deformations (Herrel et al., 2011). This can be explained by two different phenomena. First, by concentrating stresses and deforming the material in front, the wedge-shaped head reduces form drag. Second, when the eels dig, they use smaller wavelength undulations, resulting in more distributed forces in the direction of motion, and reducing stresses on the substrate. Similarly, sand lances use their wedge-shaped nose to enter the substrate via plastic deformation and then use the lateral portion of their body to undulate and push against the sand with little deformation (Gidmark et al., 2011). The same principle applies to worms using peristaltic motion: the body anchors at several places, using the substrate as a solid to provide traction, while some body parts are shrunk and pushed forth (Dorgan et al., 2016). Worms use crack propagation to move inside the ground, especially when the soil is not too soft. Peristaltic waves end with the enlarging of the tip of the front end of the worm, which acts as a wedge to crack the soil (Murphy and Dorgan, 2011; Grill and Dorgan, 2015). This wedge appears to reduce the form drag by reducing the worm’s frontal area. If the soil is too soft, it deforms rather than cracking when subjected to high stresses, and the worm moves by plastic rearrangement of the grains (Dorgan et al., 2016). To grip the soil more firmly, some worms also have chaetae on their segments, which protract during the stance phase to increase friction and retract during the forward movement (Foxon, 1936; Crane and Merz, 2017), creating a friction anisotropy. Figure 4B presents the worms’ peristaltic motion.

Mole crabs are using their legs to dig into the substrate and burrow themselves. For this purpose, they use four pairs of legs to scrape the material off the ground. During the digging motion, legs are successively extended or retracted, hence creating motion anisotropy and enabling the body to burrow (Trueman, 1970; Faulkes and Paul, 1997; Treers et al., 2022). This animal is also suspected of using fluidization, which will be described later.

##### 4.1.3.2 Material removal

**FIGURE 5:**
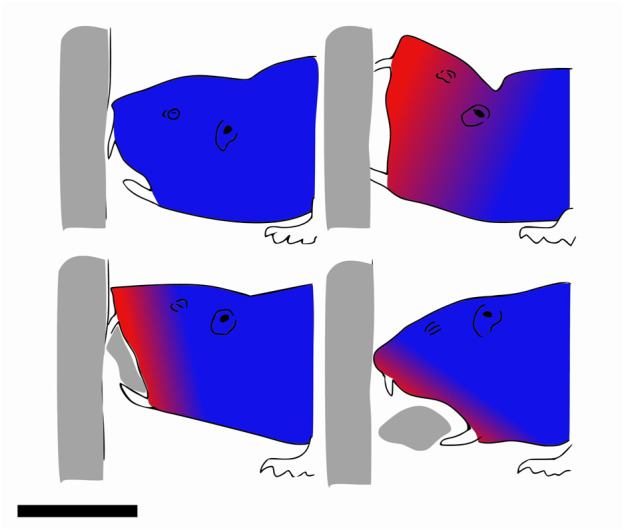
Mole rat digging a tunnel with his teeth. Red parts are the moving parts. Side view, scale bar is 3 cm. Drawn after Van Wassenbergh et al. (2017).

This strategy entails digging a tunnel to eliminate form drag on the body as it travels through the media. It cannot be used in a purely fluidic or solid medium and is, therefore, specific to soft plastic grounds. Some examples of animals using this strategy are moles (Yalden, 1966; Scott and Richardson, 2005), mole rats (Jarvis and Sale, 1971; Van Wassenbergh et al., 2017) (Figure 5), mole crickets (Zhang et al., 2011), and the arthropod *Nebalia bipes* (VANNIER et al., 1997). These animals make use of different methods of digging. Moles use their arms equipped with large paws to plastically deform the substrate and eventually remove it from the front. So do the mole crickets, while the mole rats dig using their teeth. The *Nebalia bipes* digs into unconsolidated mud by scraping the surface with its paws. Additional anisotropy is created by a microscale structure on its shell, facilitating progression through mud while hindering backward motion (VANNIER et al., 1997).

As it was seen in this section, animals progressing statically through the media are all using anisotropy, either through oscillations, surface features like chaetae, or microscales or body deformation. Some animals also take extra steps to reduce the form drag of the media, either by removing it, or by plastically deforming it using a wedge-shaped frontal end of the body.

### 4.2 Robotic analogs

As we will see in this section, the technologies used for locomotion in robotics can be classified using the same categories as the strategies derived for animals. The abstraction of the principles helps understand how technologies that may at first seem very different, can be brought together under the same principle.

Most of the robots that used discrete contacts with the medium were not designed to move on soft yielding media, but they were shown to walk on sand or mud with varying degrees of success.

The most common legged robots demonstrated on yielding substrates are hexapods. The AmphiHex (Liang et al., 2012), RHex (Li et al., 2013), and SandBot (Goldman et al., 2009) robots are all hexapods, which enable them to reduce the pressure on a single foot. However, as observed in some of the experiments (Li et al., 2009; Zhong et al., 2018), the small area and length of the feet relative to the robot’s weight do not allow an effective motion as the robot struggles in deformable media. Experiments using the SandBot (Li et al., 2009; Qian et al., 2015) showed that for very weak grounds, increasing the step frequency too much and/or reducing the material compaction leads to dramatic performance losses. The performance drop is probably due to an increase in acceleration-related vertical ground support force that leads to deeper sinkage of the legs (Li et al., 2009; Qian et al., 2012). The BasiliskBot was designed to study the effects of substrate properties on the locomotion parameters and showed that a higher sand saturation level led to increased stride length, which, in turn, increased velocity (Bagheri et al., 2017). Similar velocity drops were observed at higher stepping frequencies. A crab-like robot was built to investigate how crab dactyls could improve sand anchoring (Graf et al., 2021). It was found that, despite a clear increase in the generated anchoring forces, the use of pointed, curved dactyls reduced the locomotion speed on yielding media. Some quadruped robots were also used in yielding media: Lee et al. (2020a) demonstrated the quadruped robot ANYmal treading over natural terrains. Even though the robot hardware is not well-adapted for locomotion in these deformable terrains, robust control enables the robot to traverse ground coated with mud or snow. Using the same principle and similar hardware, the BigDog robot was demonstrated walking in mud and snow (Raibert et al., 2008).

Other experiments used a bottom–up approach, investigating how different materials or actuation strategies could affect forces and sinkage. For example, an experiment using a variable stiffness jamming foot was built to prove that using a soft deformable foot allows for less deceleration, sinkage, and pull-out forces when interacting with sand due to increased surface area and internal work (Chopra et al., 2020). Additionally, stiffening the foot after sinkage allows for more shear force to be generated. Another experiment tested different stepping parameters on muds and found that foot compliance increased the generated force on mud, and that lower speeds lead to higher forces (Godon et al., 2022).

Other robots maintained continuous contact with the ground. In Baines et al. (2022), a turtle-inspired robot that can change the shape of its appendages was designed. The appendages can be changed from legs to flippers to either walk, crawl, or swim. The turtle robot uses the crawling gait on yielding media to ensure stability and avoid stress concentration. It was shown that the cost of transport is correlated with the friction coefficient of the shell and negatively correlated with the friction coefficient of the appendages. Additionally, crawling on four flippers was more efficient than that on two flippers, showing that bio-inspired robots can be capable of outperforming their biological inspiration. A sea-turtle robot (Mazouchova et al., 2013) has been designed to mimic some aspects of the crawling locomotion on sand. It was demonstrated that crawling with a flexible wrist helped locomotion by reducing the work done on the material, and that flipper-induced lift enabled to reduce drag on the body. Additionally, a similar mudskipper robot (McInroe et al., 2016) showed how the tail could improve crawling on low-yield substrates, especially on inclines where limbs alone are not sufficient to provide thrust. A *Nereis* robot was created to explore undulatory locomotion on sand, aided with elastomer appendages (Sfakiotakis et al., 2016). This robot could move on sand thanks to the distribution of its mass over a large body. Appendages were providing the propulsive force, aided by body undulations. It was observed that, even though the joint compliance between segments reduced the average velocity, this enabled the robot to pass all the presented obstacles.

A snake robot has been created to study sidewinding locomotion (Marvi et al., 2014). Analogous to the animal, this robot deforms the sand to create lumps of sand, which then solidify and serve as a static support.

The ePaddle robot was designed to incorporate a wheel-paddle mechanism to negotiate both unstructured and yielding grounds as well as water and solid plane ground (Shen et al., 2018). On sand, the paddles dig into the ground to obtain more traction and reduce slippage.

More generally, man-made ground vehicles fit in this category. Examples include wheeled vehicles (the Sherp ATV (SHERP, 2022) and Burlak (BURLAK, 2022)) which use large, deflated wheels to spread the weight on a large surface area to reduce soil vertical deformations. In addition, the tires of these vehicles incorporate large studs to gain traction on the soil. Other examples include tracked vehicles (Ripsaw tank (RIPSAW, 2022) and the Tinger track (TINGER, 2022)), which are designed to increase friction and distribute weight. Yet, this strategy can only work if the layer of the yielding medium is shallow, or the yield stress is very high, because these vehicles do not have the buoyancy that the wheeled vehicles take advantage of. Screw-propelled vehicles have been proposed, built, and proven reliable in muddy and sandy surfaces (MudMaster (PHIBION, 2022) and RUA (Fales et al., 1971)). Typically, on a solid soil, the helix will yield the substrate and then push on it without yielding it further. On very soft ground, like dry sand or very thin and wet mud, or even water, its behavior will look more like a paddle inside a fluid.

Now let us analyze the robot prototypes using static locomotion through the medium.

A few robots have been built to move using non-reciprocal motion. For example, a sperm-inspired robot was built, mimicking the flagella oscillations of sperm and enabling locomotion in a friction-dominated medium (Khalil et al., 2016). The sandfish lizard robot has been designed to mimic the high rate undulations of the animal, and that has enabled the robot to swim in a granular substrate (Maladen R. et al., 2011). Even though studies on the animal demonstrated granular fluidization (see next section), the robot appears to take advantage only of non-reciprocal motion in a dense frictional flow. The RoboClam is inspired by the razor clam (Winter et al., 2014) and uses the clam’s dual-anchor mechanism to dig efficiently through unconsolidated media. Ortiz et al. (2019) shows a worm-inspired robot that uses peristaltic motion to move through dry granular media. Similarly, Liu et al. (2019) demonstrated a worm-inspired robot with a patterned skin that increases traction during the anchoring phase of the peristaltic movement and is retracted during the advance. A robot for planetary subsurface exploration was created and tested in a regolith simulant (Zhang et al., 2019). The robot uses a dual-anchor mechanism that enables anisotropy, similar to the alternating anchoring/forward motion observed in earthworms. When one anchor is pushing on the walls, the other one is retracted and moved in the direction of motion with a pushing module between the anchors. This motion is combined with material removal and is described in the next paragraph. A similar burrowing robot was designed and tested in soil (Omori et al., 2012). The main difference with the robot in Zhang et al. (2019) lies in the presence of four propulsion sub-units that mimic the peristaltic motion of earthworms, thereby creating motion anisotropy.

Some robots use the material removal technique to move through the medium; for example, Kobayashi et al. (2011) created a mole-inspired burrowing robot capable of moving through soft yielding soil by plastic deformation of the ground, resulting in a tunnel. The material is not dug out when boring a tunnel but rather pushed aside. The arms, using an anisotropic motion, are perpendicular to the material while progressing and parallel to the body during the swing phase. In Treers et al. (2022), a mole crab-inspired robot was built, able to dig itself through the sand by statically moving the sand from below to the top. To create larger forces during the digging phase than during the recovery motion, the legs are retracted, hence creating frictional anisotropy. Lee et al. (2020b) developed a mole rat robot that was inspired both by the mole rat’s teeth-scraping for the digging mechanism and by the mole for material removal. On top of the non-reciprocal motion, the underground drilling robot mentioned in the previous paragraph (Zhang et al., 2019) uses material removal. The body of the robot consists of an excavation module and a propulsion module, connected by a propulsion module. All three modules are screw-shaped to allow transport from the front of the robot to the back, thereby eliminating form drag. The similar reddish soil-burrowing robot (Omori et al., 2012) also uses material removal through a screw-based excavation unit. The main difference is that in the latter prototype, the material is conveyed through the body. Another lunar subsurface explorer was designed and tested on sand (Nagaoka et al., 2010a). The robot consists of a cylindrical body with a contra-rotor screw drill (CSD). The CSD is a cone on which two contra-rotating sections are responsible for loosening the regolith material and pushing it backward. The propulsion force is generated by backward displacement of the material.

As seen in this section, animals and robots use a large diversity of techniques and technologies to move through soft deformable media using its static properties. Now, let us observe the second mechanical principle used to move through such environments, dynamics-based motion.

## 5 Dynamics-based movements

To move using the dynamic properties of the medium, a body has to rely on the medium’s inertia to exert a sufficient force. To facilitate locomotion on/through a yielding material, a body can make it behave as a fluid, thereby reducing its resistance. Analogous to what was observed in the statics-based interactions, two different strategies were observed:

• Discrete contacts with the medium
• Go through the medium

We did not find any examples of dynamics-based locomotion using continuous contact with the medium in the robotics or biology literature. However, some man-made vehicles use this strategy. Examples are a full-throttle dirt bike going over a mudflat or a propeller-propelled boat powerful enough to generate thrust in mud. Although the reason for the absence of this strategy in nature or robotics could not be identified in the literature, we believe it does not provide any benefits that would justify its usage. As we will see in this section, the use of dynamics-based motion requires making use of the medium’s inertia with discrete, powerful strokes or fluidizing it. A dynamic locomotion based on continuous contact may not benefit from form drag reduction with fluidization since the major part of the body lies outside of the yielding material. This type of locomotion may not benefit from having a continuous, frenetic movement at the interface between air and yielding media either, as this would likely require substantial power to continuously expel material backward/downward. While animals sometimes use power-intensive locomotion strategies for mating, escaping, or preying, they generally tend to use energy-efficient locomotion strategies (Schmidt-Nielsen, 1997; Alexander, 2003). It is, thus, not surprising that no animal was discovered using this inefficient strategy nor that no bio-inspired robot was discovered using it, or any robot, particularly given the generally inefficient locomotion of robots (Kashiri et al., 2018).

### 5.1 Animals’ locomotion

Similar to the previous section, we will first look at locomotion strategies in animals and then at their robotic analogs.

#### 5.1.1 Discrete contacts with the medium

**FIGURE 6:**
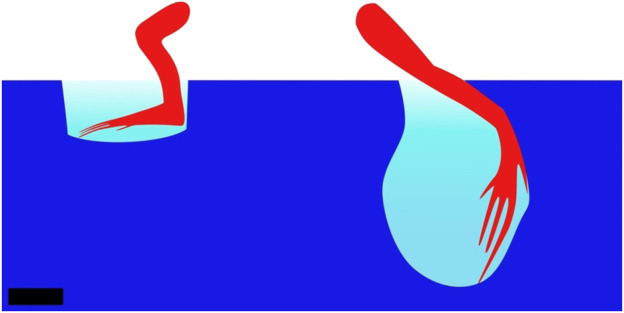
Basilisk lizard leg during the slap (left) and stroke (right) phases or a step. During the slap, the inertia of the column of water under the foot and the hydrostatic pressure generated by the column of displaced fluid above the foot generate reaction force, which enables the lizard to not sink and gain forward momentum. During the stroke phase, the same principles apply, but force is directed forward. The leg is then retracted from the water before the created air pocket collapses. Side view, scale bar is 1 cm. Drawn after Hsieh and Lauder (2004).

The basilisk lizard uses high-rate deformations to move on water and along watersides. Even though water cannot be considered a yield-stress material, the basilisk lizard shows how using high-rate deformations enables it to stay on top of a fluid. By stepping very quickly on the fluid with long digits and increasing the surface area, the lizard takes advantage of the dynamic properties of fluids. Stepping quickly on a fluid causes a column of fluid to move beneath the foot’s surface. The inertial resistance of this column of fluid enables some force to be applied on it (Hsieh and Lauder, 2004). On top of the inertial effect, hydrostatic pressure and shear resistance of the fluid, i.e., the friction induced between layers of fluid because of its viscosity, contribute to the reaction force. The latter is negligible at high Reynolds numbers such as for the basilisk lizard on water (Bush and Hu, 2006; Park et al., 2008). Figure 6 depicts the leg of a basilisk lizard during the slap and stroke phases. These forces (inertial, hydrostatic, and viscous forces) can enable animals to run on fluids. The higher the fluid density and the higher its viscosity, the easier it is to stay on the surface. This same principle can be applied to moving on soft flowable media such as mud or sand. The basilisk lizard has been observed to balance and avoid sinking into a flowable substrate by reducing its stride length as the surface hardness diminishes (Bagheri et al., 2017). *Callisaurus* lizards have been observed on sand using their foot as a paddle to generate force when sinking into the flowable material. The energy lost during frictional dissipation in the yielding material is compensated for by the upper hind muscles (Li et al., 2012). Paddling through a fluidizing medium is based on the momentum given to elements of fluid, in the same way one propels with a paddle on a boat.

**FIGURE 7:**
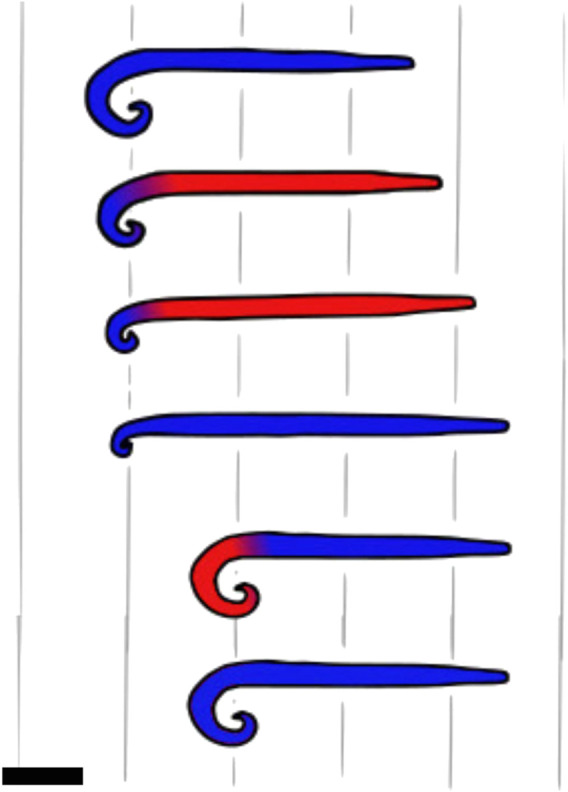
Hopping pattern of *Theristus caudasaliens*. The red parts are moving forward, while the blue ones are not. The worm jumps by bending its tail and rapidly releasing it to generate a short and powerful stroke on the medium. Top view, scale bar is 0.1 mm. Drawn after Adams and Tyler (1980).

Other animals are also using a similar effect to move on flowable materials. To propel itself, the worm *Theristus caudasaliens* makes short and powerful strokes on the ground (Adams and Tyler, 1980). An illustration is provided in Figure 7. Some blennies similarly hit the soil with their tails to jump forward (Hsieh, 2010). They also orient the wide lateral surface of their tails toward the soil to get more grip. The larger surface area leads to a larger column of fluid being pushed and also leads to distributed efforts to reduce the pressure. The arthropod *Nebalia bipes* also uses short strokes of the tail for propulsion while digging into mud. Additionally, the microscale structure on its shell is suspected to improve hydrodynamics by degenerating turbulence close to the surface of the animal, which, in turn, helps progression through mud (VANNIER et al., 1997).

#### 5.1.2 Through the medium

Some animals use high-rate deformation to fluidize the material. This strategy is used by the sandfish lizard, for example, which undulates its body to transform the sand into a fluid-like material, enabling it to swim inside the sand (Maladen et al., 2009; Goldman, 2014). The razor clam has also been described as using the fluidization of the water bottoms to burrow at depths where the forces required to dig are higher than what it produces. By agitating its shell at high speed, the clam creates pressure drops that break the walls of the tunnel, make the mud behave as a fluid, and reduce the required force to dig itself into the substrate (Trueman, 1983; Winter et al., 2012).

Worms like *Scalibregma inflatum* have also been described as using fluidization of the sand underwater by moving their bodies and appendages (Dorgan et al., 2016).

**FIGURE 8:**
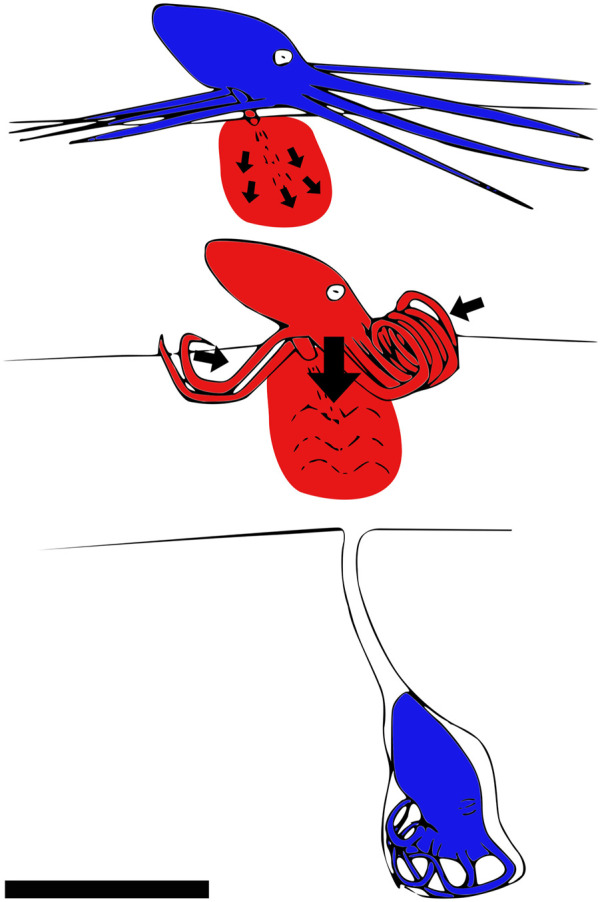
How the octopus *Kaurna stranks* digs itself under the saturated sand by using fluidization. The moving parts are shown in red. The octopus first injects water into the sediments, thereby fluidizing the medium. Then, it inserts itself into the fluidized sediment and creates a mucus-covered chimney with its tentacles to breathe. Side view, scale bar is 10 cm. Drawn after Montana et al. (2015).

Montana et al. (2015) shows how the octopus *Kaurna stranks* digs itself into the ground using fluidization by jetting water into saturated sand. It also secretes mucus to solidify the walls of the tunnel. The octopus shown in Figure 8 uses fluidization to dig itself into the sand.

Trueman (1970) showed the digging behavior of sand crabs in saturated sand, and it is speculated that the sand passes into a fluid-like state when the crabs give a high velocity to the sand particles.

### 5.2 Robotic analogs

Similar to what was observed in statics-based locomotion, robotic analogs were found using dynamics-based locomotion.

Two examples of robots were found in literature using the dynamic properties of the sand with discrete contacts. Both are hexapods using one-degree-of-freedom rotary legs. The SandBot was the first legged robot to demonstrate fluidization with rotary legs (Li et al., 2009). Experiments using this robot showed that an increase in the stride frequency and/or a decrease in soil compaction can lead to a dramatic loss of speed on a flowable substrate, probably due to its fluidization (Li et al., 2009). In this case, this was an undesired effect as it decreased the speed and efficiency. Zhang et al. (2013) demonstrated that DynaRoach, a similar rotary-leg, cockroach-inspired walking robot, that is lighter and has wider legs than the SandBot, uses static-like forces at low speeds, but transitions to hydrodynamic-like forces when legs’ rotation frequency increases, thereby increasing its locomotion speed. This means that contrary to low speeds, where hydrostatic forces balance the weight and enable sufficient tangential forces to be applied for moving, high speeds are dominated by particles’ inertia, where the robot generates forces by accelerating sand particles in the opposite direction. DynaRoach could benefit from the inertia of the particles because its wider legs enable it to increase the amount (and therefore, mass) of particles being given momentum, and its lighter body requires less momentum to gain velocity. These two examples, and particularly the latter, where the locomotion benefited from the hydrodynamic-like behavior of the medium, show how inertia can be used to step quickly on a yielding medium and accelerate particles in the opposite direction to obtain enough momentum to move. However, these examples also show us, similar to the basilisk lizard, that low weight, wide appendages, and high speeds are required. The combination of lightweight, high instantaneous power, and a large surface contact area is technically challenging and leaves few design options for a robot using this locomotion strategy. This is probably limiting the strategy’s ability to scale.

Robots making use of fluidization to move through the medium were also rare in the literature. The RoboClam has been created to mimic the razor clam’s locomotion and manages to dig with decreased energy expenditure thanks to high-rate agitations of the shell that fluidize the mud (Winter et al., 2014). Similar to the animal, the RoboClam creates pressure drops in the fluid surrounding the walls of the burrow, which leads to the fluidization of the material and reduces its resistance. It was also demonstrated that using only the fluidization motions without using the dual-anchoring motions enabled the robot to burrow under its own weight. Naclerio et al. (2018) created a robot that advances by extending its body, inspired by plant root growth. The robot grows from the tip, reducing skin friction drag because the rest of the body remains immobile relative to the ground. It also fluidizes the sand to reduce form drag by blowing air in the direction of motion. The fluidization enables the robot to reduce the penetration force into the sand by an order of magnitude, especially for higher air flows. The two examples previously mentioned show robotic devices that use fluidization to reduce penetration resistance into yielding materials, in a similar way to animals using fluidization through the medium. This differs from robots or animals using discrete, dynamic contacts with the medium, where the objective is to use the fluid’s inertia to generate thrust. In the two cases shown here, the robots were demonstrated to reach depths they could not reach without the use of fluidization. This strategy could also be used to move horizontally through the medium while using less energy. Last, the sandfish lizard robot was demonstrated to swim in the sand using mainly frictional forces (Maladen R. et al., 2011), but it appears to use fluidization locally at the tail and head, even though the contribution of this fluidization to locomotion may be limited (Ding et al., 2012).

## 6 Physical principles to move on soft deformable grounds: analyses, gaps, and discussion

The locomotion mechanisms described in previous sections often share common physical principles that facilitate the animal to negotiate yielding terrains. Those principles can be used by animals regardless of their anatomy or locomotion pattern and are, therefore, common to many species. A summary of this classification can be found in Table 2. Next, we classified the robots accordingly. This can be found in Table 3.

**TABLE 2:** Modes of locomotion used by animals: arthropods (Foxon, 1936; Trueman, 1983; Herreid and Full, 1986; Faulkes and Paul, 1997; Herreid, 2012; Kuroda et al., 2014), basilisk lizard (Bagheri et al., 2017), blennies (Hsieh, 2010), burrowing eels (Herrel et al., 2011), caterpillars (Trimmer et al., 2006), *Callisaurus lizard* lizard (Li et al., 2012), *Clarias* (Johnels, 1957), climbing perch (Davenport and Matin, 1990), cows (Phillips and Morris, 2000), (Telezhenko and Bergsten, 2005), elephants (Weissengruber et al., 2006; Panagiotopoulou et al., 2012), gastropods (Trueman, 1983), hatchling turtles (Mazouchova, 2012), humans (Lejeune et al., 1998), (McMahon and Greene, 1979), inchworms (Plaut, 2015), leeches (Dorgan, 2010), lizards (Carothers, 1986; Kohlsdorf et al., 2001; Vanhooydonck et al., 2015), lungfishes (Horner and Jayne, 2014), mole crab (Trueman, 1970; Faulkes and Paul, 1997; Treers et al., 2022), mole crickets (Zhang et al., 2011), moles (Yalden, 1966), mole rats (Van Wassenbergh et al., 2017), mudskipper (HARRIS, 1960; Van Dijk, 1960; Pace and Gibb, 2014), *Nebalia bipes* (VANNIER et al., 1997), *Nereis virens* (La Spina et al., 2007), octopus (Montana et al., 2015), *Polypterus senegalus* (Standen et al., 2016), razor clams (Trueman, 1983; Winter et al., 2012), salamanders (Edwards, 1989; Aydin et al., 2017; Vega and Ashley-Ross, 2020), sandfish lizard (Maladen et al., 2009; Goldman, 2014), sand lances (Gidmark et al., 2011), seals (O’gorman 1963), snakes (Jayne, 1986; Wake, 2001), sperm cells (Friedrich et al., 2010), *Theristus caudasaliens* (Adams and Tyler, 1980), and worms (Foxon, 1936; Grill and Dorgan, 2015; Dorgan et al., 2016; Crane and Merz, 2017)

	Static-based				Dynamic-based	
Animal	Discrete C.	Cont. C.	Through N-recip.	Through Exc.	Discrete C.	Through
Arthropods	x					
Basilisk lizards	x					
Blennies					x	
Burrowing eels			x			
Caterpillars	x					
*Clarias*		x				
Climbing perch		x				
Cows	x					
Elephants	x					
Gastropods		x				
Hatchling turtles	x	x				
Humans	x					
Inchworms	x					
Leeches	x					
Lizards	x					
Lungfish	x					
Mole crab				x		X
Mole crickets				x		
Moles				x		
Mole rats				x		
Mudskipper		x				
*Nebalia bipes*				x		X
*Nereis virens*			x			
Octopus						X
*P. senegalus*		x				
Razor clams			x			X
Salamanders	X					
Sandfish lizard						X
Sand lances			x			
*S. inflatum*						X
Seals		x				
Snakes		x				
Sperm cells			x			
*T. caudasaliens*					x	
Worms			x			

**TABLE 3:** Bio-inspired modes of locomotion used by robots: AmphiHex (Liang et al., 2012), amphibious robot turtle (Baines et al., 2022), BasiliskBot (Bagheri et al., 2017), BigDog (Raibert et al., 2008), crab-like robot (Graf et al., 2021), CSD robot (Nagaoka et al., 2010a), dynaRoACH (Zhang et al., 2013), ePaddle robot (Shen et al., 2018), inchworm robot (Zhang et al., 2019), mole crab robot (Treers et al., 2022), mole-inspired robot (Kobayashi et al., 2011), mole rat robot (Lee et al., 2020b), mudskipper robot (McInroe et al., 2016), NASA’s mini rover (Shrivastava et al., 2020), *Nereis* robot (Sfakiotakis et al., 2016), planetary subsurface explorer (PSE) (Omori et al., 2012), RHex (Li et al., 2013), RoboClam (Winter et al., 2014), sandfish robot (Maladen R. et al., 2011; Ding et al., 2012), screw-drive rover (Nagaoka et al., 2010b), SeaDog (Klein et al., 2012), sea turtle robot (Mazouchova et al., 2013), sidewinding rattlesnake robot (Marvi et al., 2014), sperm-shaped robot (Khalil et al., 2016), tetrad-screw robot (Lugo et al., 2017), tip-extending burrowing robot (Naclerio et al., 2018), and worm-inspired robot (Liu et al., 2019; Ortiz et al., 2019). Brackets describe an undesired effect.

	Static-based				Dynamic-based	
Animal	Discrete C.	Cont. C.	Through N-recip.	Through Exc.	Discrete C.	Through
AmphiHex	X					
Amphib. turtle		x				
BasiliskBot	X					
BigDog	X					
Crab-like r.	X					
CSD r.				x		
dynaRoACH	X				x	
ePaddle		x				
Inchworm r.			X	x		
Mole crab				x		
Mole r.			X			
Mole rat r.				x		
MudskipperBot		x				
NASA’s rover	X	x				
*Nereis* r.		x				
PSE r.			x	x		
RHex	X					
RoboClam			x			x
SandBot	X				(x)	
Sandfish r.			x			x
Screw-drive r.		x				
SeaDog	X					
Sea turtle r.		x				
Sidewinding r.		x				
Sperm-shaped r.			x			
Tetrad-screw r.		x				
Tip-extending r.			x			x
Worm-inspired r.			x			

### 6.1 Current state of research and research gaps

Table 3 shows that robots have been primarily developed for using the static-based ways of locomotion. Dynamic-based locomotion alone has been marginally used for robot locomotion. Of course, swimming robots have been developed for underwater environments, but no evidence has been found that they would be capable of swimming in yielding materials. Indeed, using dynamics-based locomotion in yielding materials can mean hitting it very quickly to stay on the surface. It can also mean to fluidize it with frenetic oscillations or fluid projection, both of which require a high power output.

Both discrete and continuous ground contacts have been extensively studied in statics-based locomotion strategies. This does not imply that the problem of locomotion in these environments has been solved. Indeed, the large majority of the robots presented here are not fully working solutions but instead were intended to study a specific aspect of locomotion. Each robot contributes to the comprehension of locomotion on yielding substrates. Some of these robots, for example, were aimed at testing models of yielding materials, others were testing a specific hardware or kinematic feature of a robot, while others aimed at understanding a phenomenon observed on an animal. We can also see that animals digging/burrowing statically are much less explored. This can probably be explained by the reduced fields of applicability of such robots compared to robots moving above the ground. It is also worth noting that no animals or robots are using a dynamic-based continuous contact locomotion strategy. One possible reason might be that this locomotion strategy combines high velocity with continuous drag, resulting in what appears to be a very inefficient, energy-demanding, and potentially ineffective solution.

It is important to note that not all the robots analyzed in Table 3 are explicitly bio-inspired. Indeed, some robots using principles such as force distribution or increased friction have been exploited for a long time, even in the automotive industry, and can be achieved by other means than copying the solutions from nature, for example, fat tires, tracks, and screws. Nonetheless, the abstraction level we proposed, based solely on physics-based interactions on the higher level and the nature of contacts on the second level, allows one to bring together solutions such as the crawling gait of the mudskipper and Archimedean screw-based robots’ locomotion, demonstrating the classification’s potential for use in biomimetics.

### 6.2 Discussion

This paper’s main contribution is a review of biology and robotic literature to identify locomotion principles that can be used in robot design in yielding environments. The principles are classified at two different levels, one considering the mechanical principle, the other considering the locomotion strategies to exploit that principle. The higher levels of abstraction also allow expanding the ontology to non-bio-inspired robots. It is worth noting that using abstract language and non-technical terms to describe a problem is a well-known systematic problem-solving technique proposed to avoid tunnel vision and early fixation (Al’tshuller 1999). The abstraction level used in this review proposes to classify locomotion strategies regardless of animal or robot morphology. As it is common in the biomimetics methodology, the resulting classification can, thus, assist researchers or engineers interested in locomotion on yielding grounds to easily pass from the problem domain to the solution domain (Vincent et al., 2006; Verhaegen et al., 2011; Fayemi et al., 2017). This process widens the range of potential solutions and prevents early zeroing in on a solution directly mimicked from nature, which may be suboptimal or impossible to implement. In the current case, it can assist the designer in defining the problem and proposing more diverse solutions for the problem of motion in low-yield environments by understanding general physics-based principles.

The results described in this paper have some limitations. First of all, we observed that biology literature strictly addressing the biomechanics of animals in yielding environments is very scarce, especially when compared to the papers generally addressing legged locomotion, flying biomechanics, swimming biomechanics, etc. Even in the identified papers, the focus of the paper was often on some other aspects (e.g., the behavior of the animal), and biomechanics was only very briefly described. Even when the papers focused on bio-locomotion, they often used biology terminology and methods rather than those of physics and mechanics, for example, the locomotion mechanisms were descriptive rather than mathematically formulated, and physical quantities were not measured. This lack of mathematical modeling, necessary for robotics, probably explains the frequent tendency of bio-inspired robotics research to incorporate bio-locomotion research as a preamble in the same paper. This represents an additional difficulty for roboticists trying to develop bio-inspired robots. Recently, robophysics research has started to address such topics with a more mechanics-based approach (Aguilar et al., 2016). In some cases, due to the lack of mathematical modeling in research papers, our interpretations of the physical principles are partly speculative. For example, the effect of stress timing and duration on yielding environments has received little attention in the biology literature. In such cases, we had to make assumptions on the motions of some animals based on drawings or verbal descriptions, and these assumptions could be proven inaccurate. However, the principles used for the classification follow the known laws of physics; we, therefore, believe that even if an animal was misclassified, this does not question the main contributions of this research: the general, abstracted locomotion principles, and the catalog of bio-inspired solutions. This work can still serve as inspiration and a general theoretical framework for someone who wants to design robots or understand animal locomotion principles.

The developed ontologies indicated several research gaps and opportunities for improvement. First of all, some strategies (e.g., dynamics-based discrete contacts) are scarcely addressed in the biology literature, which also limits the research opportunities in bio-inspired robotics. Principles using the static properties of yielding substrates are most commonly used by robots, and among those walking mechanisms, discrete contacts have been most widely addressed. Although continuous contact motion-based robots are developed for terrestrial and underwater environments, they are often tested on a single yielding medium in a laboratory environment. This offers the possibility of expanding the research problems using already existing robot platforms or already developed methods. Finally, because the ontologies’ higher level of abstraction is physics-based, there is no need to focus on bio-inspiration. We believe that it offers some guidelines for developing useful robots and vehicles using state-of-the-art technologies. Furthermore, recent years have witnessed advances in the modeling of interactions between the locomotors and yielding materials, particularly in dry granular materials, through the use of RFT or geometric mechanics, as discussed earlier. We hope the classification proposed here will help researchers in the field to explore similarities between different locomotion strategies and further develop the theories of locomotion in yielding environments.
